# Comparison of ActiGraph CentrePoint Insight Watch Placement on Dominant and Nondominant Wrists in Young Adults in Free-Living Conditions: Observational Validation Study

**DOI:** 10.2196/63033

**Published:** 2025-08-11

**Authors:** Daehyoung Lee, Haley Voermans-Dean, Jung Eun Lee, Jong Cheol Shin, Gregory Dominick

**Affiliations:** 1 Department of Health Behavior and Nutrition Sciences, University of Delaware, 26 North College Avenue (013 Carpenter Sports Building), Newark, Delaware, 19716, United States, 1 302 831 0762; 2 Department of Applied Human Sciences, University of Minnesota Duluth, Duluth, Minnesota, United States; 3 Palmer College of Chiropractic, Davenport, Iowa, United States; 4 Department of Community and Population Health, Lehigh University, Bethlehem, PA, United States

**Keywords:** wrist-worn accelerometry, physical activity, sedentary time, device placement, measurement

## Abstract

**Background:**

With the continuous evolution of technology, wearable accelerometers have become one of the most popular means of measuring daily physical activity (PA) levels. Despite the conventional use of the nondominant wrist as a device placement in numerous PA studies, the impact of wrist-worn accelerometer placement on PA data outcomes remains uncertain.

**Objective:**

This study aimed to examine the degree of agreement between accelerometry data collected from CentrePoint Insight Watches (CPIWs; ActiGraph) worn on the dominant and nondominant wrists of young adults in free-living conditions.

**Methods:**

Twenty-nine participants (mean age 20.2, SD 1.6 years; 23 females) simultaneously wore an ActiGraph CPIW on both dominant and nondominant wrists for 7 consecutive days during waking hours. A sampling frequency of 32 Hz and Montoye 2020 cut-points were used to categorize activity intensity based on counts per minute. Data validity criteria included (1) ≥600 minutes per day of monitor wear time for both wrists, (2) a daily wear time difference of <1% of the average wear time between the dominant and nondominant wrists, and (3) a minimum of 3 valid days of monitor wear for both wrists. Bland-Altman plots and intraclass correlation coefficient (ICC) analyses were performed to compare the accelerometry data between the two device placements.

**Results:**

Average daily monitor wear time was 789.6 (SD 86.1) minutes per day for the dominant wrist and 793.0 (SD 91.8) minutes per day for the nondominant wrist. All accelerometer variables, including sedentary time (ST), light PA, moderate-to-vigorous PA (MVPA), steps, triaxial counts, and vector magnitude (VM), showed good-to-excellent levels of reliability between the two measurements (ICC >0.88 for all; *P*<.001). Bland-Altman analysis calculated mean bias and SD between the two device placements as follows: ST (−18.8, SD 27.6 min/d), light PA (2.7, SD 15.9 min/d), MVPA (12.7, SD 26.7 min/d), steps (218.1, SD 476.6 counts/d), x-axis (99.4, SD 188.8 counts/min), y-axis (73.9, SD 147.0 counts/min), z-axis (107.6, SD 183.5 counts/min), and VM (161.2, SD 273.4 counts/min). Bland-Altman plots revealed that the upper and lower limits of agreement across most variables were considerably wide.

**Conclusions:**

Our findings partially align with previous research, demonstrating higher MVPA and step counts on the dominant wrist, while the nondominant wrist produced a higher level of ST. Despite the acceptable level of reliability between the two placements based on ICC analyses, the dominant wrist tended to produce greater outcomes as the intensity of PA increased, highlighting the need for careful consideration when determining the wear location of CPIWs and interpreting data outcomes.

## Introduction

Participation in regular physical activity (PA) contributes substantial benefits to overall health, such as weight control, brain health, bone and muscle strengthening, and emotional regulation [1,2]. For this reason, PA levels primarily serve as an informative gauge of general health status for both the public and health care practitioners [3,4]. The 2018 National PA Guidelines for Americans suggest that adults should participate in at least 150 minutes of moderate-to-vigorous PA (MVPA) or 75 minutes of vigorous-intensity PA per week, along with additional muscle-strengthening activities, to achieve health benefits [5]. Accurately quantifying PA levels is a key component in developing and analyzing public health initiatives to promote regular PA participation in the general population [6,7]. Among numerous approaches for PA measurement and estimation, accelerometer-derived outcomes not only help individuals objectively recognize their PA levels but also build foundational knowledge for organizations and communities to develop health intervention plans for specific populations [8,9].

With the continuous evolution of technology, sensor-based ecological measurement has emerged, and wearable activity trackers have become one of the most popular means of measuring daily PA levels [10]. Global health technology companies, including Apple, Garmin, and Fitbit, have revolutionized the market for commercially available activity or fitness-tracking devices by making them affordable, easily accessible, and compatible with other electronic devices and applications [11]. Despite their widespread use, commercial wearable devices have shown limited accuracy in estimating PA levels in free-living environments, largely due to relatively low intradevice reliability and limited criterion validity. Most available studies have been conducted in controlled laboratory settings, focusing primarily on basic metrics, such as step counts and distance, rather than comprehensive assessments of PA parameters [12]. Moreover, their measurement accuracy varies significantly by device brand and type, and the criterion validity of comparative data is relatively poor [12,13]. ActiGraph triaxial accelerometers (ActiGraph LLC) have been used extensively by researchers across the world to estimate various PA parameters across diverse clinical and nonclinical populations, with high reliability and validity in both laboratory and free-living conditions [14]. Although limited affordability and a lack of real-time feedback are recognized as drawbacks compared to consumer-level devices [15], ActiGraph accelerometers remain among the most widely used research-grade instruments for PA measurement [16].

Due to poor compliance of wear time during the measurement period, which often results in inaccurate classification or misrepresentation of physical behaviors (eg, overrepresentation of MVPA or underrepresentation of sedentary time [ST]), an increasing number of research studies are exploring the use of wrist-worn accelerometers instead of waist-worn devices [17]. Nonetheless, there is a lack of agreement in current literature as to how the placement of wrist-worn devices (eg, dominant vs nondominant or right vs left) affects the accuracy and interdevice reliability of accelerometer data outcomes. A few studies have used earlier models of ActiGraph triaxial accelerometers (eg, GT3X, GT3X+, and GT9X) to compare specific PA outcomes collected from both the dominant and nondominant wrists in free-living settings [18,19]. Buchan et al [18] reported no significant differences between the two device placements for wear time and MVPA; however, they monitored the behavioral outcomes during waking hours for only 1 day. Park et al [20] also compared the two device placements during waking hours in a single-day, free-living setting and found that the dominant wrist tended to yield higher step counts compared with the nondominant wrist. Interestingly, a similarly designed study using comparable data collection protocols (eg, 24-hour monitoring) revealed that there was no substantial difference in PA levels measured by ActiGraph GT3X accelerometers on both wrists [19]. A recent systematic review also examined how ActiGraph accelerometry compares to popular commercial wrist-worn devices for estimating step counts, energy expenditure, and heart rate, highlighting the dearth of validity evidence for their usability in free-living settings [13]. Few attempts have been made to investigate the impact of accelerometer placement on measurement outcomes in free-living conditions using research-grade accelerometry with a 7-day monitoring protocol, which can significantly increase the reliability of predicting and representing an individual’s habitual behaviors [21,22]. A recent study by Buchan et al [7] compared 7-day wear time between the dominant and nondominant wrists. The results suggested that the intensity gradient may be equivalent between both wrists, but average acceleration was slightly higher on the dominant wrist.

Given the increasingly dominant use of wearable technologies to monitor and estimate PA levels, it is important to clarify the discrepancy in the literature and verify the conventional use of the nondominant wrist as the preferred device placement in PA measurement studies. The objective of this study was to examine the degree of agreement between PA data collected from accelerometers worn on the dominant and nondominant wrists of young adults in free-living environments. This study used one of ActiGraph’s newest models, the CentrePoint Insight Watch (CPIW; ActiGraph LLC), which features a unique user-centered band design and high-resolution raw acceleration data that may improve participant comfort and data reliability. In addition, the relatively new, cross-validated vector magnitude (VM) count cut-points by Montoye et al [23] were used to determine and classify activity intensity, improving the practical implications of the study findings.

## Methods

### Participants

Twenty-nine young adults (mean age 20.2, SD 1.6 years; 23, 79.3% females) from a Midwestern university in the United States were recruited for this study. Flyers were posted around the university campus during the spring term, and prospective participants were encouraged to contact the research team for the eligibility screening. Inclusion criteria were as follows: (1) aged 18‐25 years, (2) ability to follow study protocols and independently provide informed consent, and (3) walking as a primary form of ambulation without the use of assistive devices. Individuals with a mobility impairment (eg, use of a wheelchair or crutches) or a history of cardiovascular disease were excluded from this study. Prospective participants were invited to an individual meeting with the research team, during which study procedures were thoroughly reviewed for all participants.

### Procedures and Instruments

Self-administered demographic and general health information was obtained from the participants with the assistance of a trained student investigator. This included date of birth, biological sex, height, weight, and hand dominance, which were used to initialize the ActiGraph CPIWs (dimensions: 1.97×1.35×0.41 in and weight: 14 g) for each participant. Prior to the accelerometry data collection, participants attended a training session on how to wear and care for the device. Specifically, the research team emphasized wearing and removing both accelerometers simultaneously and placing them high on their wrists with appropriate tightness to prevent slippage during movement or daily activities. They were asked to ensure the accelerometers were placed at the same relative positions on the dorsal aspect of their wrists, as variations in proximity to the hand could affect acceleration and activity counts. Participants were also asked to carry out their normal activities of daily living while wearing the accelerometers. To avoid the risk of water damage to the devices, participants were asked to take the devices off during any water-based activities, such as swimming and bathing, as well as during sleep. Participants wore an ActiGraph CPIW on both their dominant and nondominant wrists during waking hours for 7 consecutive days, for at least 10 hours per day. The watch displayed only the date and time without any interpretive information on participants’ PA levels, which is an intentional design feature to minimize reactivity to accelerometer-based measurement [24]. In addition, remote synchronization and notification features of the ActiGraph CPIW were disabled to avoid influencing participants’ habitual behaviors and PA engagement. ActiGraph CPIW enables PA data collection for up to 30 days without charging, and thus, participants were not instructed on how to charge the devices.

For this study, accelerometers were programmed to record acceleration data at a default sampling frequency of 32 Hz using 60-second epochs, which were subsequently converted to counts per minute (CPM) based on the Montoye et al [23] VM count cut-points to classify the activity intensity. Specifically, the CPM cut-points included sedentary (<2860 CPM), light PA (2860‐3940 CPM), and MVPA (≥3941 CPM). Accelerometer activity count of three axes (ie, vertical, horizontal, and mediolateral) is a measure that indicates quantified acceleration within a designated epoch length [25,26]. For data processing, accelerometer data were first saved in the CentrePoint portal (ActiGraph LLC) and later exported to ActiLife (version 6.14.0; ActiGraph) to validate the wear time and score the collected datasets based on the predetermined CPM cut-points. The wear criteria threshold for both wrists was set at ≥600 minutes per day, and nonwear time was defined as ≥90 minutes of consecutive zero counts with an allowance of 2-minute spike tolerance [27]. After the 7-day data collection period, participants attended a debriefing meeting with the research team to return the accelerometers. In addition, perceived PA level was assessed using a 4-category classification approach outlined in the Physical Activity Guidelines for Americans (PAGA), 2nd edition, which includes the following PA levels: inactive, insufficiently active, active, and highly active [5].

### Statistical Analysis

PA as a variable was measured using the following parameters: step counts per day, time spent at each PA intensity, including light PA and MVPA (min/d), activity counts from 3 axes, and VM CPM. VM was calculated using the following formula: |V| = √(Axis X^2^+Axis Y^2^+Axis Z^2^), to describe in-depth analyses of sampled accelerations of movement detected from both device placements. Considering that reactivity to accelerometer measurement is most pronounced at the beginning of the PA data collection [24], the first day of the 7-day accelerometry data was intentionally removed from the analyses. Data validity criteria included: (1) ≥600 minutes of monitor wear time per day for both wrists, (2) a daily wear time difference of less than 1% of the average wear time between the dominant and nondominant wrists, and (3) a minimum of 3 valid days of monitor wear for both wrists during the same periods. Of the 30 participants who completed the entire study, 29 participants met all 3 data validity criteria and were included in the analyses. One participant was excluded for having only one valid day of monitor wear. Each participant generated 3-6 valid days of monitor wear, and the total number of valid days included in the analyses was 153. We calculated the average of each data metric across valid days for each participant and compared the data outputs from the 2 device locations on the individual level. Paired samples *t* tests were performed to examine differences in PA and ST data collected from dominant and nondominant wrists. Bland-Altman plots and intraclass correlation coefficients (ICCs) were used to determine the degree of agreement between the two device placements. The ICC value ranges from 0 to 1, where <0.5=poor, 0.5‐0.74=moderate, 0.75‐0.9=good, and >0.9=excellent reliability [28]. In addition, linear regression analyses were conducted to assess the relationship between the magnitude of measurements and individual bias. The coefficient of determination (*R*^2^) was interpreted as follows: ≤0.01=small, ≈0.09=medium, and ≥0.25=large effect size [29]. As a follow-up analysis, the equivalence test, also known as the two one-sided test (TOST), was performed to determine whether the means of the two different measures were equivalent within a 5% margin and whether any found differences were clinically significant. To conclude significant equivalence within the specified margin, both one-sided tests (ie, upper and lower bounds represented as *t_1_* and *t_2_*) must reject the null hypothesis of nonequivalence (ie, unequal to zero). All statistical analyses were performed using IBM SPSS Statistics version 28, and the significance level was set at *P*<.05.

### Ethical Considerations

Written informed consent was obtained from all participants prior to their participation in this study. All participants received a US $20 prepaid debit card as an incentive at the end of their study participation. The institutional review board at the University of Minnesota approved this study (STUDY00017556).

## Results

Participant characteristics are shown in Table 1. The average weight of the participants was 63.6 (SD 8.3; males: 70.7, SD 5.1; females: 61.8, SD 8.0) kg. Except for 2 (6.9%), most participants (n=27; 93.1%) were nonsmokers at the time of study participation. Of the 29 participants, 2 (6.9%) indicated left as their dominant wrist or hand. Average daily monitor wear time was 789.6 (SD 86.1) minutes per day for the dominant and 793.0 (SD 91.8) minutes per day for nondominant wrists, respectively. The average number of valid monitor wear days was 5.3 (SD 1.3; median 6, IQR 2) days.

The descriptive accelerometry data between dominant and nondominant wrists and the results of the paired samples *t* tests are shown in Table 2. According to the ICC analyses, all accelerometer variables, including ST, light PA, MVPA, steps, triaxial counts, and VM, showed good to excellent levels of reliability between the two device placements (ICC >0.88 for all; *P*<.001; refer to Table 3).

**Table 1.** Sample characteristics (N=29).

Characteristic	Value
Age (years), mean (SD)	20.2 (1.6)
Female, n (%)	23 (79.3)
Weight (kg), mean (SD)	63.6 (8.3)
Height (cm), mean (SD)	168.3 (10.2)
BMI (kg*m^−2^), mean (SD)	22.5 (2.9)
Ethnicity, n (%)	
White	24 (82.8)
African American	0 (0)
Asian	3 (10.3)
Hispanic	2 (6.9)
Class standing, n (%)	
Freshman	4 (13.8)
Sophomore	7 (24.2)
Junior	15 (51.7)
Senior	3 (10.3)
Proximity to campus, n (%)	
On campus	5 (17.2)
Within 1 mile	16 (55.2)
Within 3 miles	6 (20.8)
Within 5 miles	1 (3.4)
More than 5 miles	1 (3.4)
Perceived PA^a^ level, n (%)	
Highly active	17 (58.6)
Active	9 (31.1)
Insufficiently active	3 (10.3)
Inactive	0 (0)

a PA: physical activity.

**Table 2.** Descriptive accelerometry data from dominant and nondominant wrists (N=29).

Variable	Dominant, mean (SD)	Nondominant, mean (SD)	Average, mean (SD)
ST^a^ (min/d)	526.9 (89.6)	545.7 (93.2)^b^	536.3 (91.1)
LPA^c^ (min/d)	154.6 (33.9)	151.9 (39.0)	153.3 (36.3)
MVPA^d^ (min/d)	108.1 (44.1)	95.4 (41.3)^b^	101.8 (42.8)
Steps (per day)	8304.6 (3337.5)	8086.1 (3193.9)^b^	8195.5 (3239.6)
x-axis (CPM^e^)	1601.6 (401.6)	1502.2 (389.9)^b^	1551.9 (395.5)
y-axis (CPM)	1740.8 (570.7)	1666.9 (572.7)^b^	1703.9 (567.9)
z-axis (CPM)	1666.8 (342.0)	1559.2 (365.4)^b^	1613.0 (355.0)
VM^f^ (CPM)	2911.1 (727.5)	2749.8 (740.8)^b^	2830.5 (732.2)

a ST: sedentary time.

b Statistically significant difference compared to dominant wrist; *P*<.05.

c LPA: light physical activity.

d MVPA: moderate-to-vigorous physical activity.

e CPM: counts per minute.

f VM: vector magnitude.

**Table 3.** Intraclass correlation coefficients between dominant and nondominant accelerometer measures (N=29).

Variable	ICC^a^^b^ (95% CI)	*F* test (*df*)	*P* value
ST^c^ (min/d)	0.97 (0.88‐0.99)	42.88 (28)	<.001
LPA^d^ (min/d)	0.95 (0.90‐0.98)	20.01 (28)	<.001
MVPA^e^ (min/d)	0.88 (0.70‐0.94)	9.23 (28)	<.001
Steps (per day)	0.99 (0.98‐0.99)	186.87 (28)	<.001
x-axis (CPM)^f^	0.93 (0.81‐0.97)	16.58 (28)	<.001
y-axis (CPM)	0.98 (0.95‐0.99)	59.46 (28)	<.001
z-axis (CPM)	0.91 (0.74‐0.96)	13.88 (28)	<.001
VM^g^ (CPM)	0.95 (0.86‐0.98)	27.85 (28)	<.001

a Average measures with a two-way mixed effects model were used.

b ICC: intraclass correlation coefficient.

c ST: sedentary time.

d LPA: light physical activity.

e MVPA: moderate-to-vigorous physical activity.

f CPM: counts per minute.

g VM: vector magnitude.

**Figure 1.**
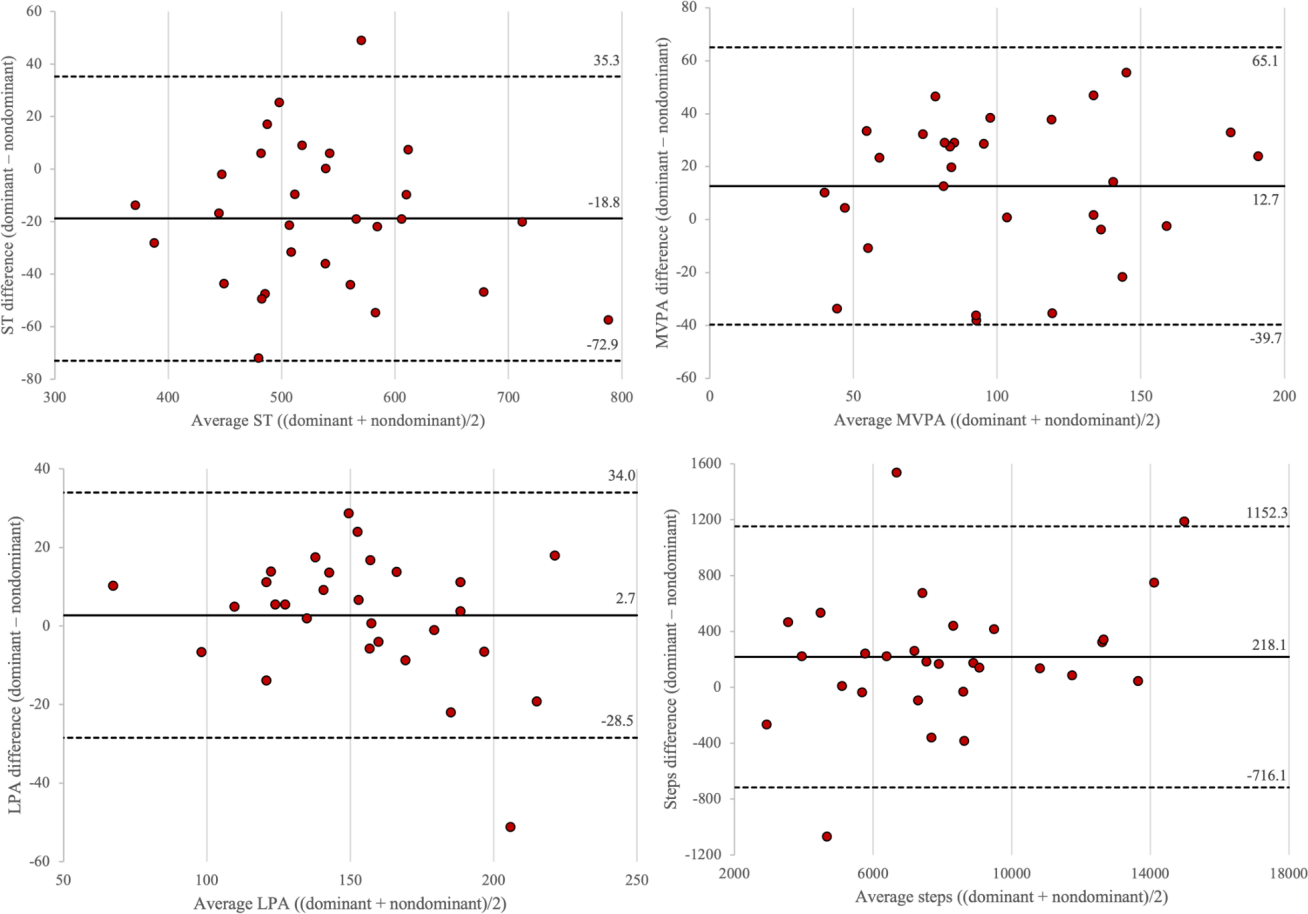
Bland-Altman plots were used to assess data agreement between the dominant and the nondominant wrists in measuring sedentary time, light physical activity, moderate-to-vigorous physical activity (min/d), and steps per day. Each red circle represents the individual-level difference between the two device placements for the 29 participants. Two dotted black lines indicate the limits of agreement (mean difference ±1.96× SD of the differences), and a solid black line indicates the mean bias between the two measures. LPA: light physical activity; MVPA: moderate-to-vigorous physical activity; ST: sedentary time.

Bland-Altman analysis calculated mean bias and SD between the two device placements as follows: ST (−18.8, SD 27.6 min/d), light PA (2.7, SD 15.9 min/d), MVPA (12.7, SD 26.7 min/d), steps (218.1, SD 476.6 counts/d), x-axis (99.4 SD 188.8 counts/min), y-axis (73.9, SD 147.0 counts/min), z-axis (107.6 SD 183.5 counts/min), and VM (161.2, SD 273.4 counts/min). Figures1  2 display individual-level biases with upper and lower limits of agreements. It is estimated to have proportional bias when there is a linear tendency in the discrepancies between the two measures, which is often represented by the slope of the regression or *R*^2^ that significantly differs from zero [30]. Proportional bias was considered present in steps (*R*^2^=0.091) and light PA (*R*^2^=0.105), given the medium effect sizes for the regression coefficients, coupled with the progressive divergence in the generated plots. However, follow-up TOSTs with a 5% equivalence margin revealed that none of the accelerometry variables showed statistically significant equivalence between the means of two device placements: *t_1_* and *t_2_* for ST=0.33 and −1.90; light PA=1.08 and −0.52; MVPA=1.59 and 0.68; steps=0.73 and −0.22; x-axis=1.70 and 0.21; y-axis=1.06 and −0.08; z-axis=2.03 and 0.29; and VM=1.57 and 0.10 (all *P*>.05).

**Figure 2.**
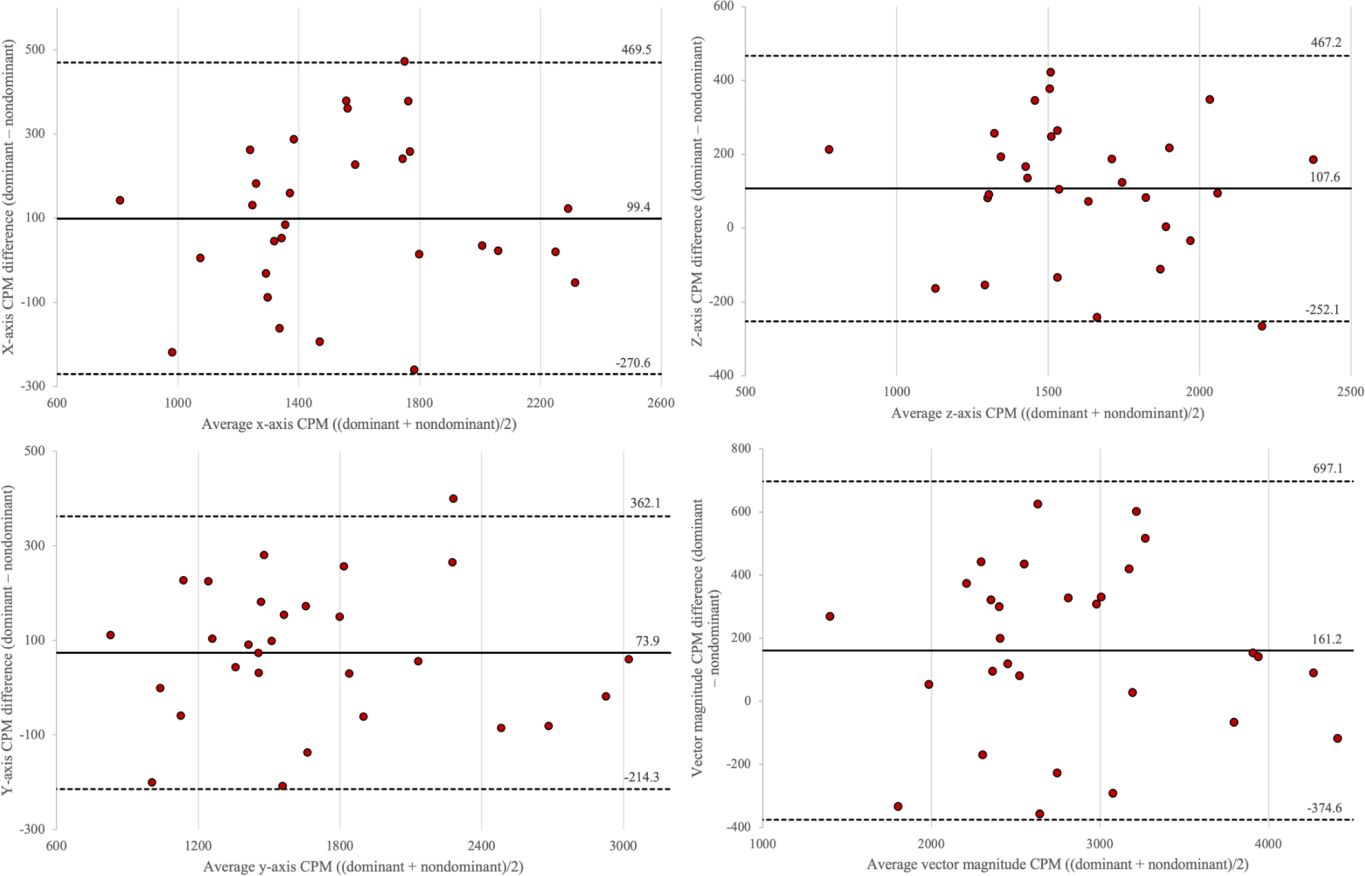
Bland-Altman plots were used to assess data agreement between the dominant and the nondominant wrists in measuring x, y, and z axes, as well as vector magnitude (counts per minute). Each red circle represents the individual-level difference between the two device placements for the 29 participants. Two dotted black lines indicate the limits of agreement (mean difference ±1.96× SD of the differences), and the solid black line indicates the mean bias between the two measures. CPM: counts per minute.

## Discussion

### Principal Findings

The objective of this study was to examine the degree of agreement between accelerometry data collected from ActiGraph CPIWs worn on the dominant and nondominant wrists of young adults in free-living conditions. This study comprehensively compared accelerometry variables, including ST, light PA, MVPA, and steps, as well as activity counts of 3 axes and VM, between the two device placements. Despite statistically significant differences observed through paired *t* tests, all outcome variables between the two placements showed good-to-excellent levels of comparability. However, the limits of agreement were considerably wide across all variables except ST, highlighting the need for cautious interpretation of these mixed findings.

Overall, previous research comparing PA data collected from the dominant and nondominant wrists revealed mixed results with limited consistency. A recent study examined PA data from ActiGraph GT9X accelerometers worn on both wrists for a 1-day data collection period and reported that the dominant wrist consistently produced higher step counts than the nondominant wrist in free-living conditions [20]. This result aligns with our findings, wherein the dominant wrist produced relatively higher outcomes of MVPA, light PA, and steps than the nondominant wrist. Conversely, an earlier study used a nearly identical protocol and found that device placement had no impact on the average daily PA outcomes, regardless of the axis examined in free-living environments [19]. Our study partly supports this finding, as all accelerometer variables, including the 3 axes and VM, exhibited excellent ICC values between the two measurements. However, proportional bias was considered present in steps (*R*^2^=0.091) and light PA (*R*^2^=0.105). This implies that the dominant wrist tended to produce higher step counts as the average step level increased, while the opposite trend was predicted for light PA. Given that 7 consecutive days of monitor wear are recommended for investigating representative and predictable PA behaviors, differences in wear periods (eg, 1 d vs 7 d) and the use of different ActiGraph models (eg, GT9X and GT3X vs CPIW) may contribute to the inconsistent results [31,32].

As explained by the paired *t* test results, MVPA, steps, and all axis-related variables showed statistically significant differences between the two placements, in which the dominant wrist produced relatively higher levels of outcomes compared to the nondominant wrist. Interestingly, the opposite result was observed in ST, whereby the nondominant wrist produced a higher level of outcome than the dominant wrist. Montoye et al [33] compared research-grade accelerometers (ie, GENEActiv; Activinsights Ltd) worn on both wrists for measuring PA and sedentary behavior. Their findings underscored higher sensitivities and specificities (eg, 97%) in predicting sedentary behavior as well as low-intensity activities using the left wrist-worn accelerometer (left hand was indicated as the nondominant hand among most participants), compared to the right wrist-worn accelerometer. Meanwhile, the right wrist-worn accelerometer (mostly the dominant wrist among participants) produced significantly lower sensitivity and specificity for MVPA, which was overly misclassified as light PA due to underestimation of MVPA. Similarly, Fraysse et al [34] used GENEActiv accelerometers to compare the classification accuracy between dominant and nondominant wrists in free-living environments. While their sample consisted of older adults (ie, ≥70 years of age), significantly higher ST and lower MVPA were linked to the dominant wrist compared to the nondominant wrist. Once again, these mixed results may be attributed to the use of different accelerometer brands (eg, ActiGraph vs GENEActiv) that can result in varying raw acceleration outputs [35]. More comprehensive cross-validation studies are needed to determine the degree of agreement among a variety of research-grade accelerometer brands and models according to specific movement contexts and populations.

It should be noted that the paired *t* test considers only the overall bias of two measures; thus, this approach may not fully address how rejecting the null hypothesis corresponds to the degree of agreement between the two measurements [36]. As such, we implemented follow-up TOSTs with an equivalence margin of 5% to determine whether the observed difference between the two device placements is clinically significant. None of the included variables rejected the null hypothesis of nonequivalence for both one-sided tests, indicating insufficient evidence to declare statistically significant equivalence between the two placements within the specified margin. In line with the TOST results, ICC values for all accelerometer measures indicated good-to-excellent correlation (>0.88), suggesting strong agreement between the two placements. Moreover, Bland-Altman plots of all variables showed that the individual differences between the dominant and nondominant wrists mostly fell within the limits of agreement, which highlights the comparable estimations of variables between the two placements [37]. However, given the volume of average accelerometry outcomes (ie, ST=536.3 min/d, light PA=153.3 min/d, and MVPA=101.8 min/d), the discrepancy between the two measurements may not be at the acceptable level. The mean bias for MVPA (12.7 min/d) was comparable to that for ST (−18.8 min/d), indicating lower interdevice comparability in estimating MVPA. Wrist-worn devices are often recommended to capture lower-intensity activities, especially those mainly involving upper-body movements such as household chores [38]. Therefore, our findings emphasize the practical comparability (eg, sedentary-to-light activities vs MVPA) between the two device placements in free-living conditions, in which the intensity of the activity determines the degree of agreement or discrepancy between the two placements: the higher the intensity of PA, the greater the discrepancy in outcomes between the dominant and nondominant wrists.

The current and previous findings underscore the need for cautious interpretation of ICC and Bland-Altman plots when comparing the PA data of multiple measurements [39,40]. It is critical to note that although the mean bias for most accelerometry variables may seem marginal, the limits of agreement between the two measurements were considerably wide for PA variables, spanning over 104.8 minutes per day range for MVPA (−39.7 to 65.1 min/d), nearly 2000 steps per day for walking steps (−716.1 to 1152.3 steps/d), and more than 1000 VM CPM per day (−374.6 to 697.1 CPM/d). A similar result was observed by Pfister et al [39], in which the research team examined the comparability between the waist-worn ActiGraph GT3X+ and the thigh-worn activPal3 (PAL Technologies Ltd). While the ICCs for all PA and ST variables between the two devices were generally high and consistent, statistically significant—albeit small—differences in MVPA were found between the GT3X+ VM, vertical axis, and activPal3 at the group level. Further, the limits of agreement between the two accelerometer types were generally wide at the individual level, restricting the interchangeable use of these devices [39]. Regardless of the ICC analysis results, the degree of agreement may need to be determined by the overall mean bias in comparison to the range of the limits of agreement.

When it comes to the objective measurement of physical behaviors, the anterior waist or hip has been a conventional placement in earlier studies. Karaca et al [41] compared step counts measured by wGT3X-BT (ActiGraph) in five different body locations, including both right and left wrists, and found that the waist and right upper arm showed low mean absolute percentage error compared to other placements. In addition, waist-worn accelerometers tended to provide more accurate estimates of sedentary behavior that engaged the lower extremities exclusively, compared to wrist-worn devices [42]. However, the shift to using wrist-worn accelerometers over the past decade has demonstrated relative advantages over standard waist-worn devices. Despite the lower-performing algorithm compared to waist or hip location and a lack of criterion validity, particularly in free-living settings, wrist-worn devices can enhance participant acceptability given their familiarity with a wristwatch and enable increased data collection within 24-hour movement and sleep studies [43,44]. The use of the wrist as a device placement is often preferred over the waist because wrist-worn devices are generally less intrusive and often result in higher compliance with study protocol [45,46]. Although there is a lack of scientific consensus on cut-points for categorizing activity intensities in wrist-worn accelerometers, including CPIW, it is understood that devices on the nondominant wrist are likely to ensure higher measurement accuracy than those on the dominant wrist in free-living settings [33]. Given the high variability of movement and participants’ unfamiliarity with wearing devices on the dominant wrist, the nondominant wrist may be a more reliable choice for assessing levels of PA and ST in real-world settings.

### Limitations and Strengths

It is important to acknowledge the methodological limitations that may have affected the interpretation of our findings. The use of wrist-worn accelerometers may lead to underestimation or overestimation of PA levels, as they are limited in detecting nonambulatory activities (eg, cycling and yoga) and distinguishing between varying postures (eg, sitting vs standing) [38]. This limitation should be considered when interpreting PA estimates derived from wrist-based measurements. Although it was possible for participants to wear the accelerometers in the water, we intentionally advised them to remove the monitors during water activities and sleep to minimize potential malfunction or improper device positioning, which may have underestimated their PA levels and the discrepancies between the two measures. Longer device wear time, especially during unstructured movements such as taking a shower or pool-based activities, could increase the likelihood of movement discrepancies between the two limbs. According to our follow-up survey, 1 participant consistently wore the monitors and engaged in water-based PA despite the instructions from the research team. Sensitivity analyses excluding this participant revealed that their inclusion had a minimal impact on the results. Without detailed logs of specific activities of daily living, such as brushing teeth, washing dishes, and driving, it is difficult to systematically examine differences related to hand dominance. Another limitation of this study is the use of the nondominant wrist-specific cut-points to classify activity intensities for both wrist placements. While dominant wrist–specific cut-points have been proposed using alternative metrics such as Euclidean Norm Minus One (ENMO) [47], these may not be directly comparable due to fundamental differences in data processing and calibration methods. The lack of validated CPM-based cut-points for the dominant wrist in adults highlights the need for further calibration studies to improve comparability across wear locations between the two wrists. Finally, the sample recruited in this study may also have introduced potential bias. Most participants in this study were women with relatively high levels of PA participation, as many were college students majoring in Exercise and Rehabilitation Sciences. This group included a few student-athletes who regularly engage in intensive training sessions, which may not be representative of average young adults. Prior studies suggest that there exists gender- and age-related variability in arm movement patterns and movement efficiency. Specifically, females tend to exhibit significantly less arm swing asymmetry, while males show more lateralized, asymmetric arm movements during walking [48]. Aging is also associated with reduced arm swing amplitude and less efficient arm movement coordination [49]. Thus, our sample of young adults may not accurately capture the variability in wrist-worn accelerometer–based PA estimates that could frequently occur in older populations. We acknowledge the growing importance of using open-source, monitor-independent metrics, such as ENMO, mean amplitude deviation, and Monitor-Independent Movement Summary, to enhance reproducibility and cross-study comparability in accelerometry research [50-52]. While Migueles et al [47] demonstrated that ENMO may yield greater agreement across wrist placements and proposed dominant wrist-specific cut-points, future research is warranted to directly compare these emerging metrics with VM counts across diverse device placements and evaluate their implications for interpreting accelerometry outputs in surveillance and intervention studies. Post hoc power analyses revealed that both the paired *t* tests and equivalence tests were underpowered, with estimated power ranging from 6% to 47% across primary accelerometry outcomes (eg, ST, MVPA, and steps). As such, these findings should be interpreted with caution given the limited sample size.

Despite the limitations, this study demonstrates notable strengths. First, this study used a standard 7-day data collection protocol in free-living conditions to provide more transferable knowledge for determining the use of the nondominant wrist as device placement. Moreover, the use of a user-friendly, relatively new CPM cut-point method [23] to classify ST and PA intensities is a major study strength. Past PA measurement studies have been interpreted with caution due to the misuse of the cut-points that were developed for waist or hip locations, different age groups (eg, adults vs children), different axial outputs (eg, uniaxial vs triaxial), or structured laboratory settings [53]. Our study findings, which leveraged the cross-validated cut-points, provide practical implications for future studies that adopt research-grade wrist-worn accelerometers.

### Conclusions

This study aimed to examine the degree of agreement between accelerometry data collected from ActiGraph CPIWs worn on the dominant and nondominant wrists of young adults in free-living environments. The current findings align partially with previous research, demonstrating higher step counts, MVPA levels, and axis-related outcomes on the dominant wrist, while the nondominant wrist produced a higher level of ST compared with the dominant wrist. Although we observed excellent levels of reliability based on ICC across most accelerometry outcomes, the intervals between the upper and lower limits of agreement were considerably wide, particularly in steps and MVPA, along with the potential proportional bias in steps and light PA. It is estimated that the intensity of PA determines the degree of discrepancy between the dominant and nondominant wrists (eg, the dominant wrist tends to produce greater outcomes as the intensity of PA increases). These findings emphasize caution in interpreting ICC and Bland-Altman plots when comparing PA data from multiple wear locations. Taken together, the nondominant wrist could offer a reliable option for measuring physical behaviors in free-living environments; however, potential underestimation of MVPA and overestimation of ST should be considered when interpreting data outcomes and selecting the device placement.

## Abbreviations

CPIW: CentrePoint Insight Watch
CPM: counts per minute
ENMO: Euclidean Norm Minus One
ICC: intraclass correlation coefficient
MVPA: moderate-to-vigorous physical activity
PA: physical activity
PAGA: Physical Activity Guidelines for Americans
ST: sedentary time
TOST: two one-sided test
VM: vector magnitude
